# Apples-to-Apples: Age-Sex Standardisation of Public Chest X-ray Datasets

**DOI:** 10.7759/cureus.97260

**Published:** 2025-11-19

**Authors:** Amr Badawy, Maiar Elhariry, Amrit Chirrimar, Ashrit Chohan

**Affiliations:** 1 General Surgery, Midlands Metropolitan University Hospital, Birmingham, GBR; 2 Hospital-Based Medicine, Midlands Metropolitan University Hospital, Birmingham, GBR; 3 Acute Medicine, Sandwell and West Birmingham NHS Trust, Birmingham, GBR

**Keywords:** ai and machine learning, artifical intelligence, big data analytics, big data analytics and machine learning, dataset bias, health informatics big data, radiology, radiology research

## Abstract

Background

Public chest radiograph datasets are widely used for model development and benchmarking, but differences in patient demographics can inflate apparent between-dataset differences in disease label prevalence.

Objective

To quantify the proportion of NIH ChestX-ray14 versus CheXpert prevalence differences that is explained by age and sex alone.

Methods

A cross-sectional analysis of NIH ChestX-ray14 (n=112,120 studies) and CheXpert (n=223,413) databases was performed. Sex was harmonised to Male/Female and age was categorised as 0-17, 18-39, 40-59, 60-79, and ≥80 years. Five shared labels were assessed: consolidation, atelectasis, pleural effusion, edema, and cardiomegaly. For CheXpert, label uncertainty (−1) was treated as negative in the primary analysis. For each label, we calculated crude prevalence with Wilson 95% confidence intervals and compared datasets using a two-proportion z-test. We then performed direct standardisation by reweighting CheXpert age-sex strata to the NIH age-sex distribution and reported the reduction in the crude prevalence gap attributable to age-sex adjustment.

Results

Crude prevalence was higher in CheXpert than NIH for all labels (all p<0.001). After age-sex standardisation, CheXpert prevalence decreased for every label, indicating that demographics account for a substantial share of between-dataset differences. For consolidation, the crude gap of 1.96 percentage points (6.12% vs 4.16%) decreased to a standardised gap of 1.47 percentage points (CheXpert standardised 5.63% vs NIH 4.16%), representing approximately a 25% reduction. For atelectasis, the gap declined from 4.85 to 2.84 percentage points (41% reduction approx.). For pleural effusion, the gap declined from 28.10 to 19.03 percentage points (32% reduction approx.). For edema, the gap declined from 21.70 to 14.78 percentage points (32% reduction approx.). For cardiomegaly, the gap declined from 9.45 to 6.55 percentage points (31% reduction approx.). Across labels, age-sex standardisation explained approximately 25% to 40% of the crude prevalence differences.

Conclusion

A simple age-sex standardisation step explains a large proportion of apparent label prevalence differences between NIH ChestX-ray14 and CheXpert. Routine reporting of standardised prevalence alongside crude estimates and demographic composition can improve fairness and interpretability in cross-dataset benchmarking and reduce the risk of attributing demographic composition effects to labelling or model performance.

## Introduction

Publicly available chest radiograph datasets underpin a large proportion of contemporary research in artificial intelligence (AI) for healthcare, enabling model development, benchmarking, and secondary analyses at scale [1]. Two of the most widely used resources are NIH ChestX-ray14 and CheXpert, each comprising hundreds of thousands of studies with accompanying label metadata derived from radiology reports or labelling pipelines [2,3]. While such datasets have accelerated progress, methodological concerns persist about cross-dataset comparability. Apparent differences in disease prevalence across datasets can reflect underlying differences in case mix, clinical setting, and acquisition protocols rather than true epidemiological variation, with downstream implications for model performance estimates and external validity [4].

Demographic composition is a particularly salient driver of prevalence differences. Age and sex distributions influence the frequency of common chest radiograph findings such as consolidation, atelectasis, pleural effusion, edema, and cardiomegaly [5]. If two datasets differ materially in their age-sex structure, crude (unadjusted) prevalence contrasts will conflate demographic effects with dataset-specific factors (for example, label-generation rules or inpatient versus outpatient capture) [6]. Despite this, many dataset descriptions and benchmarking studies report crude rates without standardisation, making it difficult to disentangle demographic composition from other sources of variation. CheXpert additionally encodes label uncertainty (−1), which, if handled inconsistently, can further widen or narrow crude contrasts in ways that are not attributable to biology or imaging practice [5].

Direct standardisation is a simple, transparent epidemiological tool that addresses this problem by reweighting stratum-specific (here, age-sex) rates from one dataset to the demographic distribution of a reference dataset [7]. This yields an adjusted prevalence that answers a clinically meaningful question: what would the prevalence be if both datasets had the same age-sex mix? Applying direct standardisation to public radiology datasets can clarify how much of an observed crude gap is explained by demographics alone, and how much remains to be explored in terms of labelling processes, clinical pathways, or image acquisition differences [7].

In this study, we compare crude prevalence for five shared labels: consolidation, atelectasis, pleural effusion, edema, and cardiomegaly, between NIH ChestX-ray14 and CheXpert. We then perform age-sex standardisation of CheXpert to the NIH distribution and quantify the reduction in the crude prevalence gap attributable to demographics. Our objective is to elucidate, using public metadata, the extent to which age and sex account for cross-dataset prevalence differences. Second to this, we advocate for routine reporting of standardised alongside crude metrics in dataset audits and model benchmarking studies.

Our primary objective is to quantify how much of the between-dataset prevalence difference is explained by age and sex via direct standardisation.

## Materials and methods

Study design

A cross-sectional audit of two public, de-identified chest radiograph datasets was performed to quantify the extent to which age and sex account for between-dataset differences in label prevalence. No patient contact or linkage was performed, and no imaging pixels were analysed; all analyses used accompanying metadata CSV files [2,3].

Data sources

We used the official metadata for NIH ChestX-ray14 (Data_Entry_2017) and the CheXpert train metadata (train.csv), both available under their respective data use terms. These files include per-study age, sex, view information, and label fields. CheXpert label columns are coded as 1 (positive), 0 (negative), and −1 (uncertain). NIH labels are provided as a pipe-delimited string in the “Finding Labels” column (e.g., “Cardiomegaly|Edema”).

Labels and variable definitions

Five labels present in both datasets were analysed: consolidation, atelectasis, pleural effusion, edema, and cardiomegaly. For NIH ChestX-ray14, each label was converted to a binary indicator using exact-match regular expressions against the pipe-delimited “Finding Labels” field. For CheXpert, per-label helper variables were created as binary indicators, treating 1 as positive and 0 as not positive. In the primary analysis, CheXpert uncertainty (−1) was treated as not positive (i.e., mapped to 0). Age was categorised a priori into 0-17, 18-39, 40-59, 60-79, and ≥80 years. Sex values were harmonised to Male/Female; Unknown was excluded from the primary analysis.

Exclusions due to missing demographics were negligible: in CheXpert, three records were excluded for unknown sex and two for missing age; in NIH, 0 records were excluded for either field.

Outcomes

The primary outcomes were (i) crude prevalence (proportion of positive studies) for each label within each dataset and (ii) age-sex-standardised prevalence for CheXpert after direct standardisation to the NIH age-sex distribution. Secondary outcomes were the crude gap (CheXpert crude prevalence minus NIH crude prevalence), the standardised gap (CheXpert standardised prevalence minus NIH crude prevalence), and the proportionate reduction of the crude gap attributable to standardisation, calculated as (crude gap − standardised gap)/crude gap.

Direct standardisation

For each dataset, label-specific prevalence was first computed within age-sex strata. NIH strata counts supplied the standard weights: for stratum s, weight ws = Ns / ΣsNs, where Ns is the number of NIH studies in stratum s. CheXpert standardised prevalence was then calculated as Σs (ps,CheX × ws), where ps,CheX is the CheXpert prevalence in stratum s. Weights were derived once from NIH and applied identically to each label.

We estimated 95% confidence intervals for the age-sex standardised prevalences using a bootstrap at the stratum level. In each bootstrap draw, for every age-sex stratum we simulated a number of positives from a binomial distribution with size n_i and probability equal to the observed proportion of positives in that stratum. We then recomputed the standardised prevalence using the fixed NIH weights. Repeating this 20,000 times, we took the 2.5th and 97.5th percentiles of the simulated standardised prevalences as the 95% confidence interval. Strata with n_i = 0 contribute no information.

Statistical analysis

Crude prevalence was reported with Wilson 95% confidence intervals [8]. Between-dataset crude differences were assessed using a two-proportion z-test [9] (reporting z and two-sided p) with two-sided significance at α=0.05. Given that standardised estimates are weighted composites rather than independent sample proportions, no hypothesis test was applied to standardised prevalences; these were summarised descriptively via the standardised gap and the percentage reduction in the crude gap. Analyses were executed in Google Sheets (Google LLC, Mountain View, CA, USA) using transparent formulae (e.g., ARRAYFORMULA, LOOKUP/REGEXMATCH for parsing, and SUMPRODUCT for standardisation). No imputation was performed. Because this is an exploratory dataset audit with very large sample sizes, no adjustment was made for multiple comparisons [10].

Sensitivity analyses

Two prespecified sensitivities were performed: (i) exclusion of all rows with CheXpert uncertainty (−1) for a given label (listwise per label) and (ii) alternative age binning (e.g., <60 versus ≥60 years) to assess robustness of the standardised results. Findings were compared qualitatively to the primary analysis.

Licensing and access

NIH ChestX-ray14 [2] and CheXpert [3] are free for non-commercial research use; we accessed the official metadata under their respective terms and cite the source articles accordingly. No proprietary scoring systems were used; all computations were performed in Google Sheets.

## Results

Cohort characteristics

The analysis included 112,120 studies from NIH ChestX-ray14 and 223,413 from CheXpert. Age and sex were available for both datasets after harmonisation. The age-sex distribution differed between datasets; NIH contained relatively fewer older adults and small absolute differences in the male-female mix compared with CheXpert. The full age-sex breakdown used for standardisation is provided in Table 1.

**Table 1:** Age–sex distribution by dataset (NIH ChestX-ray14 and CheXpert) n denotes the number of studies and “%” the within-dataset row percentage (totals may differ from 100% due to rounding). NIH percentages define the standard weights used to directly standardise CheXpert dataset to NIH dataset. Data derived from NIH ChestX-ray14 and CheXpert under research-use licences [2,3].

Age group	Sex	NIH n	NIH %	CheXpert n	CheXpert %
0–17	F	2233	1.99%	3	0.00%
0-17	M	3008	2.68%	0	0.00%
18–39	F	13515	12.05%	12640	5.66%
18-39	M	17179	15.32%	18736	8.39%
40–59	F	22272	19.86%	27266	12.20%
40-59	M	26865	23.96%	42008	18.80%
60–79	F	10249	9.14%	33825	15.14%
60-79	M	15664	13.97%	53169	23.80%
≥80	F	511	0.46%	17043	7.63%
≥80	M	624	0.56%	18723	8.38%

Crude prevalence across datasets

Crude (unadjusted) prevalence was higher in CheXpert than NIH for all five labels. Consolidation was 6.12% in CheXpert versus 4.16% in NIH; atelectasis was 15.16% versus 10.31%; pleural effusion was 39.98% versus 11.88%; edema was 23.75% versus 2.05%; and cardiomegaly was 11.93% versus 2.48%. Wilson 95% confidence intervals were narrow in both datasets owing to large sample sizes and did not overlap between datasets for any label. Two-proportion z-tests confirmed that all crude differences were statistically significant (p<0.001 for each label). Crude counts, proportions, 95% confidence intervals, and p-values are presented in Table 2.

**Table 2:** Crude prevalence in NIH ChestX-ray14 and CheXpert with 95% CIs and p-values Crude (unadjusted) prevalence with Wilson 95% confidence intervals; p-values and z statistics from a two-proportion z-test with pooled variance. Unknown sex and missing age were excluded from denominators.

Label	NIH n	NIH positives	NIH prevalence (95% CI)	CheXpert n	CheXpert positives	CheXpert prevalence (95% CI)	p-value (two-proportion z-test; z statistic)
Consolidation	112120	4667	4.16% (4.05-4.28)	223413	13682	6.12% (6.03-6.22)	<0.001 (z=23.57)
Atelectasis	112120	11559	10.31% (10.13-10.49)	223413	33862	15.16% (15.01-15.31)	<0.001 (z=38.71)
Pleural Effusion	112120	13317	11.88% (11.69-12.07)	223413	89314	39.98% (39.77-40.18)	<0.001 (z=166.62)
Edema	112120	2303	2.05% (1.97-2.14)	223413	53058	23.75% (23.57-23.93)	<0.001 (z=159.70)
Cardiomegaly	112120	2776	2.48% (2.39-2.57)	223413	26649	11.93% (11.79-12.06)	<0.001 (z=91.31)

Age-sex standardisation

**Figure 1:**
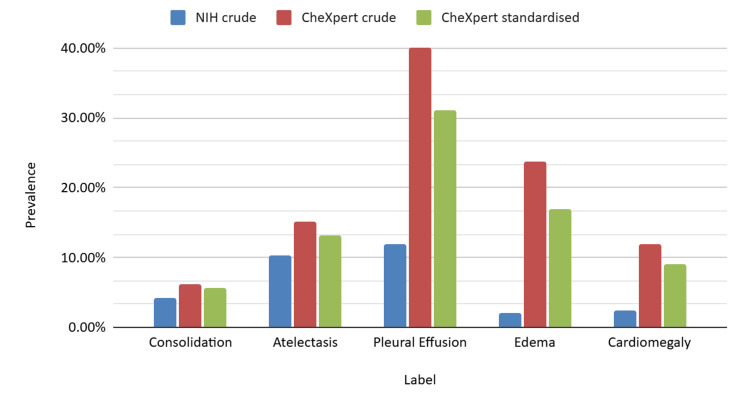
Crude vs Age-Sex-Standardised Prevalence by Radiological Label Clustered bars show NIH crude, CheXpert crude, and CheXpert prevalence after direct age–sex standardisation to the NIH distribution for five radiological labels; confidence intervals are reported in Table 2.

After direct standardisation of CheXpert to the NIH age-sex distribution, prevalence decreased for every label, indicating that demographic composition explains a substantial fraction of the crude between-dataset differences. The CheXpert standardised prevalence was 5.66% for consolidation (versus NIH 4.16%), 13.21% for atelectasis (versus 10.31%), 31.07% for pleural effusion (versus 11.88%), 16.91% for edema (versus 2.05%), and 9.08% for cardiomegaly (versus 2.48%). Corresponding standardised gaps (CheXpert standardised minus NIH crude) were 1.5, 2.9, 19.19, 14.86, and 6.60 percentage points, respectively. Expressed as reduction of the crude gap attributable to demographics, the gaps narrowed by approximately 25% for consolidation (from 1.96 to 1.5 percentage points), 41% for atelectasis (4.85 to 2.9), 32% for pleural effusion (28.10 to 19.19), 32% for edema (21.70 to 14.86), and 31% for cardiomegaly (9.45 to 6.60). These standardised estimates and gap reductions are summarised in Table 3; a visual comparison of crude versus standardised prevalence for each label is shown in Figure 1.

**Table 3:** Crude versus age–sex–standardised prevalence Crude prevalences for each label in NIH and CheXpert alongside CheXpert prevalences after age–sex standardisation to the NIH distribution. The crude gap is CheXpert minus NIH; the standardised gap is CheXpert (standardised) minus NIH. Gaps are in percentage points (pp); gap reduction is the proportion of the crude gap explained by standardisation.

Label	NIH crude %	CheXpert crude %	CheX Standardised to NIH	Crude Gap (pp)	Standardised gap (pp)	Gap reduction
Consolidation	4.16%	6.12%	5.66%	1.96 pp	1.50 pp	25.00%
Atelectasis	10.31%	15.16%	13.21%	4.85 pp	2.90 pp	40.02%
Pleural Effusion	11.88%	39.98%	31.07%	28.10 pp	19.19 pp	32.30%
Edema	2.05%	23.75%	16.91%	21.70 pp	14.86 pp	31.90%
Cardiomegaly	2.48%	11.93%	9.08%	9.45 pp	6.60 pp	30.70%

Age-sex standardisation yielded precise estimates (Table 4); for example, consolidation 5.69% (95% CI 5.58 to 5.81), atelectasis 15.52% (15.39 to 15.64), pleural effusion 39.18% (39.04 to 39.32), edema 23.05% (22.92 to 23.18), and cardiomegaly 11.09% (10.99 to 11.19).

**Table 4:** Age sex standardised prevalences with 95% bootstrap confidence intervals (CheXpert to NIH)

Label	Standardised prevalence (%)	95% CI	B
Consolidation	5.69	5.58 to 5.81	20000
Atelectasis	15.52	15.39 to 15.64	20000
Pleural Effusion	39.18	39.04 to 39.32	20000
Edema	23.05	22.92 to 23.18	20000
Cardiomegaly	11.09	10.99 to 11.19	20000

## Discussion

Principal findings

In this cross-sectional audit of two widely used public chest radiograph datasets, NIH ChestX-ray14 and CheXpert, we found that age-sex composition explains a substantial proportion of observed between-dataset differences in label prevalence. Crude (unadjusted) prevalences were higher in CheXpert than NIH for all five shared labels examined. After direct age-sex standardisation of CheXpert to the NIH distribution, the prevalence gaps narrowed consistently, by approximately one-quarter to two-fifths depending on the label, yet did not disappear entirely. For example, in the case of edema, the gap narrowed by 31.9%, meaning that approximately 68% of the crude difference remains unexplained by age and sex. 

Interpretation and implications

The consistent attenuation of gaps following standardisation indicates that the age-sex structure of a dataset is not a mere descriptive characteristic but an important determinant of apparent disease frequency. This matters for benchmarking and external validation: if one dataset simply contains more older patients or a different male-female mix, crude comparisons will overstate true differences in label prevalence and, by extension, may affect threshold selection, calibration, and performance estimates for machine learning models trained or evaluated on those datasets [11]. Our results support routine reporting of both crude and age-sex-standardised rates in dataset descriptions and model evaluation studies, as well as transparency about how uncertain labels (e.g., CheXpert −1) are handled, because uncertainty handling can shift crude contrasts independently of biology or imaging practice [12].

The residual gaps after standardisation are also informative. They suggest that factors beyond basic demographics contribute meaningfully to prevalence differences. Plausible contributors include differences in clinical setting (for example, proportion of inpatient/critical care examinations), acquisition characteristics (AP versus PA projections, prevalence of portable imaging), and the specifics of label generation (natural language processing rules, radiologist adjudication, and the treatment of uncertainty) [13]. These influences are not mutually exclusive and may interact. Our analysis cannot apportion the residual gap among these mechanisms, but it clarifies that any downstream comparison of model performance across datasets should account for both demographic composition and dataset-specific processes.

Relation to prior work

Prior studies have highlighted variability in public radiology datasets, including label noise, cohort construction choices, and site or workflow effects that can limit generalisability [14-16]. Several papers have recommended reporting demographic summaries and stratified performance, but standardisation has been less frequently applied in this context despite its long-standing role in epidemiology [17]. To our knowledge, few dataset audits have quantified, using only public CSV metadata and a no-code workflow, how much of a cross-dataset prevalence gap is explained by demographics alone and presented the remainder as a standardised gap suitable for interpretation alongside crude estimates. Our findings therefore extend existing guidance by demonstrating a lightweight, reproducible approach that any group can adopt.

Strengths and limitations

A strength of this study is its simplicity and reproducibility: all analyses were conducted using publicly available metadata and spreadsheet formulae, avoiding barriers related to code execution or protected imaging data. The approach is transparent, easily audited, and readily extended to other strata (for example, view position) when metadata permit. The large sample sizes yield precise estimates with narrow confidence intervals.

Several limitations warrant consideration. First, we relied on the variables available in the public CSVs; more granular clinical or acquisition metadata (for example, patient location, clinical indication, device type) were not available, limiting the scope of adjustment. Secondly, NIH labels are derived from a pipe-delimited string and CheXpert labels include an explicit uncertainty class; although we pre-specified handling of uncertainty and performed sensitivity checks, differences in labelling pipelines may still contribute to residual gaps.

Practical recommendations

For dataset curators, we recommend publishing age-sex standardised prevalence alongside crude rates and providing sufficient metadata to enable standardisation by additional factors (for example, projection) where feasible. For model developers and reviewers, we recommend reporting stratified performance by age and sex and considering standardised operating points when comparing across datasets.

Future work

Future analyses should investigate the residual gaps after demographic standardisation by incorporating additional metadata (for example, view position, patient location, and device type) and by examining label-generation differences explicitly. When image data access and governance permit, replication using pixel-level models may clarify how demographic and non-demographic factors jointly affect both label prevalence and downstream performance. Extensions to other public datasets and modalities would help generalise these conclusions and inform best-practice reporting standards.

## Conclusions

In summary, a simple age-sex standardisation markedly reduced apparent differences in radiological label prevalence between NIH ChestX-ray14 and CheXpert. Across five findings, demographics explained roughly a quarter to two-fifths of crude cross-dataset gaps, demonstrating that composition is a major and tractable driver. Residual differences remained, consistent with label-generation, case-mix, and acquisition factors beyond basic demographics. Routine reporting of standardised alongside crude prevalence will improve fairness and interpretability in radiological dataset benchmarking. This should be taken into consideration when using large datasets to train AI models.
